# Detection of Chlamydia trachomatis-DNA in synovial fluid: evaluation of the sensitivity of different DNA extraction methods and amplification systems

**DOI:** 10.1186/ar2864

**Published:** 2009-11-21

**Authors:** Julia Freise, Iris Bernau, Sabine Meier, Henning Zeidler, Jens G Kuipers

**Affiliations:** 1 Division of Pneumology, Hannover Medical School, Carl-Neuberg Straße 1, Hannover, 30625, Germany; 2 Division of Anaesthesiology, Diako Hospital, Gröpelinger Heerstraße 406 - 408, Bremen, 28239, Germany; 3 Division of Immunology and Rheumatology, Hannover Medical School, Carl-Neuberg Straße. 1, Hannover, 30625, Germany; 4 Rheumatologikum, Rathenau-Straße 13-14, Hannover, 30159, Germany; 5 Division of Rheumatology, Rotes Kreuz Krankenhaus, St.-Pauli-Deich 24, Bremen, 28199, Germany

## Abstract

**Introduction:**

Polymerase chain reaction (PCR) and ligase chain reaction (LCR) are used in research for detection of Chlamydia trachomatis (C. tr.) in synovial fluid (SF). However there is no standardized system for diagnostic use in clinical practice, therefore this study aimed at determining the molecular biology method best suited to detect C. tr. from SF.

**Methods:**

SF samples were spiked with C. tr. elementary bodies (EB) and human peripheral blood monocytes (PBMo) persistently infected with C. tr. in vitro to evaluate the sensitivity of different molecular biology methods and assays. Five different DNA-extraction methods were tested: 1) Alkaline lysis, 2) QIAex II Gel Extraction Kit^®^+ CTAB, 3) Chelex^®^-extraction, 4) QIAmp Tissue Kit^® ^and 5) QIAmp DNA Stool Kit^®^. All DNA extracts were subjected to 5 different DNA amplification systems to detect C. tr.- DNA in the spiked SF samples: two C. tr. -omp1-- directed PCR, one C. tr.-plasmid-PCR, one C. tr. -16s RNA directed PCR, and one commercially available LCR (LCX^®^, Abbott laboratories).

**Results:**

In SF samples spiked with C. tr.-EB and with C. tr.-PBMo, alkaline lysis, detecting 1 C. tr.-EB/ml SF, 0,1 C. tr.-PBMo/ml SF and QIAmp gel extraction kit^®^+ CTAB detecting 0,1 C. tr. -EB/ml SF, 1 C. tr.-PBMo/ml, respectively, allowed most sensitive detection of the organism in combination with the C. tr.- omp1-(152 bp) PCR. Sensitivity decreased in all methods after storage of the DNA of C. tr.- dilution series at -20°C for 4 months by at least one log phase.

**Conclusions:**

The sensitivity to detect C. tr.- DNA from SF is highly dependent on the DNA extraction method and the detection system applied. Alkaline lysis as well as the QIAmp Gel extraction kit^® ^+ CTAB in combination with C. tr.- omp1 - (152 bp) PCR evolved as the most sensitive methods to identify C. tr. in serial dilutions.

## Introduction

Chlamydia-induced arthritis (CIA) is the most frequent form of reactive arthritis (ReA) in western countries [1]. The hallmark of CIA is that the synovitis eliciting bacteria persist intraarticularly in very low quantities but cannot be cultured from synovial fluid (SF) [2,3]. Initially, immunofluorescence studies and RNA hybridization of synovial specimens were the first methods demonstrating intra-articularly persisting *Chlamydia trachomatis *[4,5]. Subsequently, from numerous reports PCR emerged as a very promising tool for the identification of *C. trachomatis *in the SF of patients with CIA and related diseases [1,6-15]. Moreover, PCR should overcome the limitations of clinical, urogenital, and serologic diagnosis of this form of ReA [16].

We previously investigated which DNA extraction methods provide the best template for PCR analysis of DNA from SF samples [8,17] as well as for synovial tissue [9]. Our results are consistent with those of other groups that noted the relevance of optimized template preparation for SF as well as for synovial tissue [18]. At present no standardized approach for *Chlamydia*-directed PCR has been described.

The aim of the present study was to define a standardized and optimized test system to evaluate clinical SF samples for *C. trachomatis *DNA in routine laboratory analysis. To address this issue we analyzed SF using spiked SF samples and human peripheral blood monocytes (PBMO) persistently infected with *C. trachomatis in vitro *in serial dilutions to investigate which template preparation methods provide the best amplification substrate for each different assay type. We also tested four different PCR systems and one commercially available ligase chain reaction (LCR) protocol in use for urogenital samples in order to determine the most sensitive system to detect chlamydial DNA from SF. The two systems best suited for detection of *C. trachomatis *was applied to clinical samples of SF (data submitted elsewhere).

## Materials and methods

### Ethical approval

Before initiation of the study ethical approval was obtained by the ethical committee of Hannover Medical School, Germany.

### Synovial fluid samples

During diagnostic or therapeutic sterile arthrocentesis from knee effusions of patients with rheumatoid arthritis or osteoarthritis, SF was collected without additives. Informed written consent of each patient was obtained before storage of SF. SF was tested for the negativity of *C. trachomatis *DNA prior to serial dilutions. Samples were stored at -20°C for between one and two weeks until further processing.

### Preparation of *Chlamydia*

*C. trachomatis *elementary bodies (EB) (serovar K) were cultured in Hep-2 cells as previously described [19]. Serovar K was chosen because it causes urogenital tract infection and has been shown to cause ReA. EB were purified in a discontinuous gradient of Urografin^® ^(Schering, Berlin, Germany) by ultracentrifugation, as described by Schmitz and colleagues [19]. EB were then resuspended in 1 ml sucrose phosphate buffer (0.01 M sodium phosphate, 0.25 M sucrose, 5 ml glutamic acid pH 7.2; Sigma, St. Louis, MO, USA) and stored at -80°C. Each preparation was analyzed by titration on Hep-2 cells and subsequent indirect immunoperoxidase assay and then adjusted to a concentration of 2 × 10^7 ^infection forming units (IFU)/ml. IFU represent the number of infective *Chlamydia *given in each sample. The *C. trachomatis *EB stock was diluted 100 fold, aliquoted and stored at -80°C. For each assay one aliquot was thawed and further diluted in 0.9% NaCl containing 0.5 mg/ml BSA for serial dilutions in *C. trachomatis-*negative SF samples.

### Serial dilution of *Chlamydia *in synovial fluid

SF samples were spiked with known numbers of *C. trachomatis *EB as previously described [9,10]. Briefly, aliquots of purified *C. trachomatis *EB were thawed and diluted to 20, 30, 40, 60, and 80 IFU/μl. Three slides were made from each dilution and each was analyzed by immunofluorescence to determine the number of *Chlamydia *EB/IFU in each dilution; the murine anti-major outer membrane protein (MOMP) monoclonal antibody used in these determinations was from the Micro-Trak system (Syva Corp, Palo Alto, CA, USA). Samples were analyzed using an epifluorescence microscope (Leitz, Wetzlar, Germany). On average, six particles corresponded to 1 IFU in each dilution (slope = 6, r^2 ^= 0.45; *P *= 0.0001). EB in known numbers were added to 1 ml SF in 10-fold decreasing numbers ranging from 10^3 ^to 10^-3 ^*C. trachomatis *EB/ml SF. One ml of SF containing no added *C. trachomatis *EB was processed in each experiment as a negative control. After addition of *C. trachomatis *EB to SF each sample was centrifuged at 60,000 g for 30 minutes at 4°C. The resulting SF cell pellet was further processed by the different DNA extraction methods described below.

### Serial dilutions of monocytes infected with *Chlamydia*

Human peripheral monocytes were prepared from healthy volunteer blood samples by the standard method, as previously described [20,21]. These monocytes were infected with *C. trachomatis *serovar K at a multiplicity of infection of 5:1 (*i.e*. 5 *Chlamydia trachomatis *EB/1 monocyte). Infected cells were analyzed by immunofluorescence to determine the number of infected monocytes in each preparation; the murine MOMP monoclonal antibodies used in these experiments was again from the Micro-Trak system. Samples were analyzed with an epifluorescence microscope. On average, 0.1% (mean 0.0967%, standard deviation (SD) 0.0037) of monocytes was infected in each preparation analyzed. At 10 days post infection, the cells were harvested and serially diluted in 10-fold decreasing steps in *C. trachomatis-*negative SF in a concentration ranging from 10^3 ^to 10^-3 ^*C. trachomatis *PBMO/ml SF. After addition of *C. trachomatis *PBMO to SF each sample was again centrifuged at 60,000 g for 30 minutes at 4°C. The resulting SF cell pellet was further processed by the different DNA extraction methods as described below.

### DNA preparation methods

Total DNA was prepared from SF spiked with *C. trachomatis *EB and *C. trachomatis *PBMO by each of five different methods; 5 μl of each DNA preparation was used for PCR and LCR analysis, respectively.

#### Method 1

Alkaline lysis was performed as described by Priem and colleagues [22]. Briefly, SF pellets were resuspended in 1 ml 1 M PBS, pH 7.0 and repelleted. Alkaline lysis was performed by overlaying the pellets with 75 μl of 50 mM NaOH in a 1.5 Eppendorf reaction tube. Samples were vortexed vigorously, spun down briefly and heated at 95°C for 15 minutes. Subsequently, neutralization was achieved by adding 12 μl 1 M Tris-HCl (pH 7.0). A 5 μl sample of the solution was either immediately subjected to PCR or LCR analysis or stored at -20°C for four months until repetition of analysis.

#### Method 2

The Qiaex II gel extraction kit^® ^+ Cetyltrimethylammoniumbromid (CTAB) was used for method 2. The Qiaex principle is based on a commercial DNA purification kit with CTAB-modification supplied by Qiagen (Hilden, Germany); preparations were performed according to the manufacturer's instructions and as described by Kuipers and colleagues [17]. SF pellets were incubated in the supplied digestion buffer (0.1 M NaCl, 1 mM EDTA, 10 mM TRIS HCl, pH 8, 0.5% Tween 20) containing proteinase K (100 μg/ml) and incubated at 56°C over night. To the samples 20 μl 5 mM NaCl was added and samples were mixed thoroughly followed by addition of 18 μl CTAB solution and incubation for 10 minutes at 65°C. Then, 140 μl chloroform (Baker, Deventer, the Netherlands) was added and samples were mixed for at least 30 seconds and subsequently centrifuged at 16,000 g for four minutes at room temperature. DNA was isolated using Qiaex II Gel Extraction Kit^® ^+ CTAB and resuspended in Tris-EDTA buffer. The Qiaex principle is based on the adsorption of DNA to silica gel particles in high salt. 5 μl of DNA solution were used immediately for PCR or LCR analysis and one aliquot of each sample was stored at -20°C for four months until repetition of PCR or LCR analysis.

#### Method 3

Chelex^® ^(Biorad, Hemel Hempstead, UK) involved DNA extraction as previously described by Wilkinson and colleagues [23] and according to the manufacturers instructions. In summary, SF pellets were digested by addition of 50 μl (150 IU) hyaluronidase (Sigma, St. Louis, MO, USA) over night at 55°C and then spun to clear. After incubation, 100 μl 10% Chelex^® ^solution was added and thoroughly mixed. Samples were then centrifuged for 10 minutes at 15,000 g and 5 μl of the resulting supernatant was used for immediate PCR or LCR analysis or stored at -20°C for four months until further PCR analysis.

#### Methods 4 and 5

QIAmp tissue kit^® ^(method 4) and QIAmp DNA Stool kit^® ^(method 5) consisted of commercially available DNA extraction kits supplied by Qiagen (Hilden, Germany); preparations were performed according to the manufacturer and as described by Branigan and colleagues [10]. SF pellets were incubated at 55°C over night in the supplied digestion buffer containing proteinase K. DNA was isolated by silica columns supplied according to the manufacturer's protocol and then eluted. Per sample, 5 μl were used for PCR or LCR analysis and remaining aliquots were stored at -20°C for four months until repetition of amplification analysis. Method 4 and 5 differ in the contents of the added extraction buffers. Exact contents of the buffers supplied are subject to patent of Qiagen (Hilden, Germany) and not known to the authors.

Five independent serial SF dilutions of *C. trachomatis *EB and *C. trachomatis *PBMO in 10 fold decreasing *C. trachomatis *concentrations ranging from 10^3 ^to 10^-3 ^*C. trachomatis *EB/ml SF and *C. trachomatis *PBMO/ml SF, respectively, were performed for each DNA extraction method. Samples were considered positive when both duplicates were detected to be positive in the subsequent PCR analysis. In each assay negative controls containing pure water as well as pure SF in the spiking assays were analyzed as negative controls. For positive controls, DNA from pure *C. trachomatis *EB were used in each sample analysis round and in each spiking assay at a concentration of 10^5 ^*C. trachomatis *EB/ml SF.

Amplifications using the five different systems described above were performed immediately after DNA extraction. DNA aliquots from serial dilution assays extracted by alkaline lysis, Qiaex gel extraction kit^® ^+ CTAB, Qiagen tissue kit^® ^and Qiagen stool kit^® ^were stored at -20°C for four months and were subjected to the most sensitive amplification system, PCR 1, again to determine stability of DNA. DNA extraction by Chelex^® ^was the least sensitive method and was therefore not reanalyzed after storage. PCR analysis was performed in duplicates. A sample was considered positive when both aliquots were detected to be positive in the subsequent PCR analysis.

### PCR and LCR analysis

Template DNA from SF EB and SF *C. trachomatis *PBMO as prepared by all previously described DNA extraction methods was subjected to PCR using four independently developed PCR primer sets and the commercially available Abbott LCX^® ^(Abbott, Abbott Park, IL, USA). Primer system number 1 (Table 1) was first described by Bobo and colleagues [24] and targets the *C. trachomatis *major outer membrane protein (*omp1*) gene; all assays were performed using the conditions described by Kuipers and colleagues [17]. Primer system number 2 targets (Table 1) a different sequence in the *C. trachomatis omp1 *gene and was developed by Gérard and colleagues [9], and the conditions were described in several papers [4,5,9,17]. Primer set number 3 (Table 1) targets a sequence within the plasmid genome of *C. trachomatis *and conditions were used as first described by Wilkinson and colleagues [23]. Primer set number 4 (Table 1) was developed by Bas and colleagues and targets a 16s RNA sequence within the chlamydial genome [18]. The LCX^® ^system (amplification system number 5) used for the present studies was the standard commercial kit supplied by Abbott Laboratories (Abbott Park, IL, USA) and targets the 7 kbp plasmid sequence in the *C. trachomatis *genome; LCR assays were performed according to the manufacturer [8].

**Table 1:** Summary of evaluated amplification methods, target on chlamydial genome, product size and references of primer sequences

	Target	Product size	Primer sequences	Application in Germany
PCR 1	*omp-1 *(152 bp)	152 bp	Bobo and colleagues [24]	Kuipers and colleagues [17]

PCR 2	*omp-1 *(739 bp)	739 bp	Gérard and colleagues [27]	Freise and colleagues [9]

PCR 3	Plasmid	402 bp	Wilkinson and colleagues [23]	M. Rudwaleit, Benjamin Franklin Hsp., Berlin

PCR 4	16sRNA	141 bp	Bas and colleagues [18]	

LCR	Plasmid	LCX^®^	Abbott	Kuipers and colleagues [17]

LCR = ligase chain reaction; omp-1 = major outer membrane protein 1.

All PCR systems employed are nested PCR systems, for product sizes see Table 1.

All PCR amplifications were carried out in an Eppendorf thermal cycler (Eppendorf, Hamburg, Germany) and primers used were synthesized by MWG Biotech (Ebersberg, Germany). LCX^® ^analysis was performed in an LCR thermal cycler; patent of (Abbott, Abbott Park, IL, USA). The oligonucleotides used by the LCR kit were supplied by the manufacturer. Purified water and *C. trachomatis *DNA of 10^7 ^*C. trachomatis *EB/ml SF was used for negative and positive controls, respectively.

Visualization of amplification products was performed by 2% agarose gel electrophoresis and ethidium bromide staining under ultraviolet light. A sample was considered positive if there was a visible amplification product of correct length, with correct negative and positive controls. The product identity of PCR 1 was confirmed by hybridization using the digoxigenin hybridization protocol from Boehringer (Ingelheim, Germany) in combination with Dyna Beads (Dynal, Hamburg, Germany) for all analyzed samples. Hybridization was performed according to the manufacturer's protocol.

**Figure 1:**
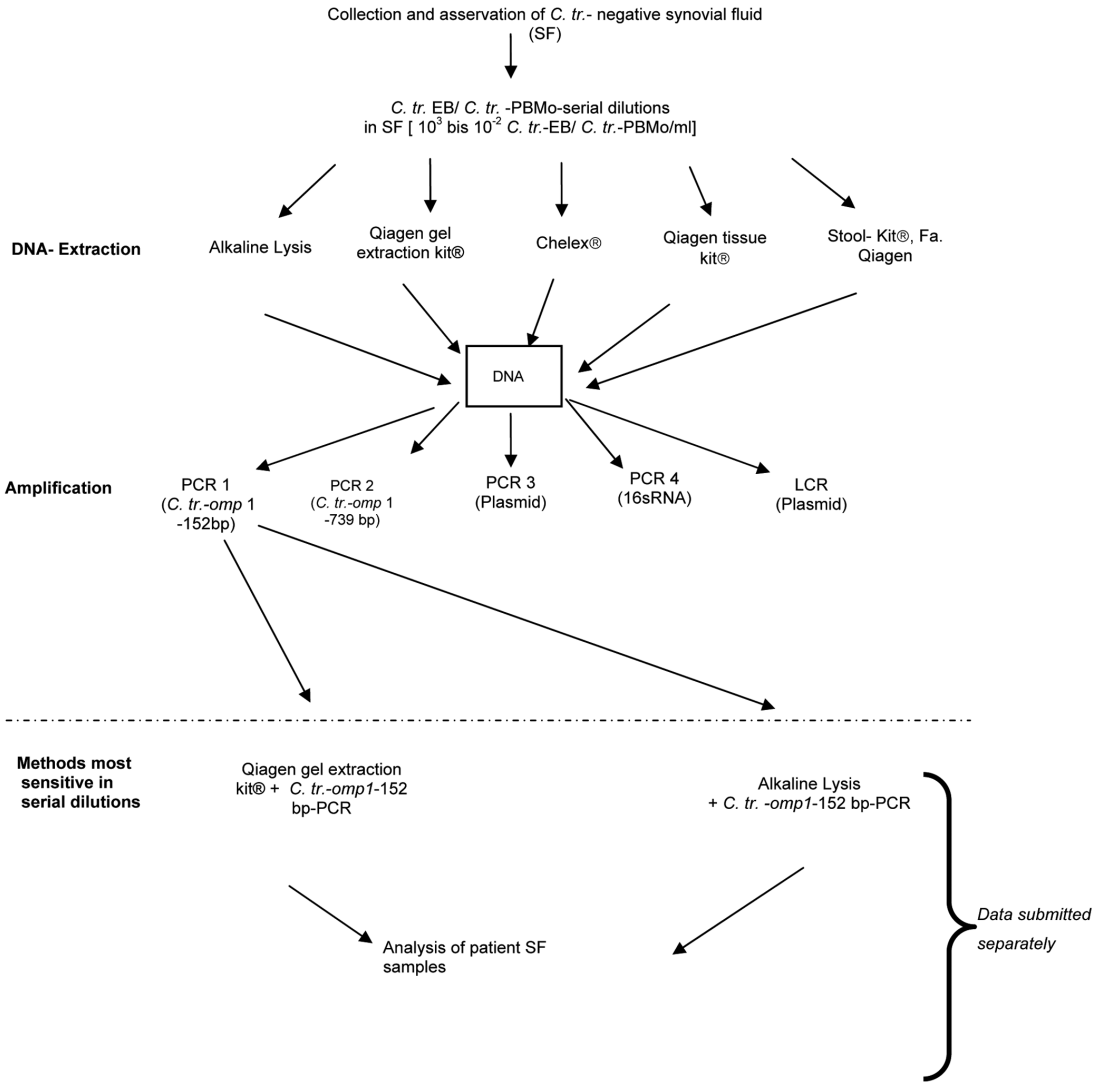
Algorithm of sample analysis. bp = base pairs; *C. tr*. = *Chlamydia trachomatis*; EB = elementary bodies; LCR = ligase chain reaction; PBMO = peripheral blood monocytes; PCR = polymerase chain reaction; SF = synovial fluid.

Figure 1 summarizes the above described algorithm of sample analysis.

### Statistical analysis

Definition of the number of EB relative to IFU was conducted by standard regression analysis. The number of *C. trachomatis *EBs and *C. trachomatis *PBMOs measured by immunofluorescence were the basis for determining sensitivity. For the PCR and LCR assays, sensitivity was defined as reproducibly detected lowest number of measured *C. trachomatis *EB/ml SF and *C. trachomatis *PBMO/ml SF. For comparison, sensitivity is given for each method as the number of *C. trachomatis *EB/ml SF and *C. trachomatis *PBMO/ml SF. Determination of statistical significant difference between the sensitivities determined for the different extraction methods using the five amplification methods was performed by the Kruskal-Wallis test, followed by the Mann-Whitney U test. A value of *P *≤ 0.05 was considered significant in all such analyses.

## Results

### Sensitivity of *Chlamydia*-directed PCR and LCR testing for *C. trachomatis *EB DNA as a function of template preparation

**Figure 2:**
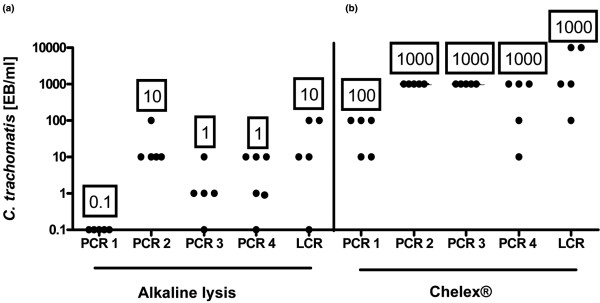
Results of PCR analysis of *Chlamydia trachomatis *EB in synovial samples following DNA extraction by (a) alkaline lysis and (b) Chelex^®^. On the y-axes concentration of *Chlamydia trachomatis *elementary bodies (EB)/ml are given. Each point on the graph indicates the detection limit of one serial dilution analysis. Numbers in boxes represent lowest reproducible detection limit of *Chlamydia trachomatis *EB/ml in synovial fluid.

Highest sensitivity (0.1 *C. trachomatis *EB/ml SF) was achieved with the Qiaex II Gel Extraction Kit^® ^+ CTAB followed by alkaline lysis, Qiagen Tissue Kit^® ^and QIAmp DNA Stool Kit^®^, which detected repeatedly 1 *C. trachomatis *EB/ml SF in combination with PCR 1. The Chelex^® ^DNA extraction method was least sensitive, detecting repeatedly 100 *C. trachomatis *EB/ml SF in combination with PCR 1. Figure 2 visualizes the raw data of alkaline lysis as the most and Chelex^® ^as the least sensitive DNA extraction method. All other detection systems achieved equal or lower sensitivities in combination with the five DNA extraction methods investigated (Table 2). In particular, PCR 3 achieved equal detection limits as PCR 1 in combination with DNA extraction by the QIAmp tissue kit^® ^(1 *C. trachomatis *EB/ml SF) and alkaline lysis (1 *C. trachomatis *EB/ml SF). PCR 4 also detected equal *C. trachomatis *EB/ml SF in combination with alkaline lysis (1 *C. trachomatis *EB/ml SF).

**Table 2:** Sensitivity of PCR for the detection of *Chlamydia trachomatis *in synovial fluid depending on DNA extraction method and primer system used

Amplification system	1 Alkaline Lysis	2 Qiaex II gel extraction kit^®^	3 Chelex^®^	4 Qiagen Tissue kit^®^	5 Qiagen Stool kit^®^
	(*C. trachomatis *EB/ml SF)	(*C. trachomatis *EB/ml SF)	(*C. trachomatis *EB/ml SF)	(*C. trachomatis *EB/ml SF)	(*C. trachomatis *EB/ml SF)
PCR 1	1	0.1	100	1	1
*C. trachomatis*	(0.1-10)	(1-100)	(10-100)	(1-10)	(1-100)
*omp *1	M1	M 1	M 100	M 1	M 100
Statistical analysis	* 2,5	* 1,4		* 2	* 1

PCR 2	10	1000	1000	10	10
*C. trachomatis*	(10)	(1-1000)	(100-1000)	(0.1-10)	(10-1000)
*omp *1	M 10	M 1000	M 1000	M10	M 10
Statistical analysis	* 2	* 1,4,5		* 2	* 2

PCR 3	1	100	1000	1	100
Plasmid	(0.1-10)	(10-1000)	(1000)	(1-10)	(10-1000)
	M 1	M 100	M 1000	M 1	M 100
Statistical analysis	* 2,3,5	* 1,3,4,5	* 1,2	* 2,3	* 1,2,4

PCR 4	1	10	1000	10	100
16 sRNA	(0.1-10)	(10-1000)	(10-1000)	(0.1-10)	(10-100)
	M 1	M 100	M 1000	M 10	M 10
Statistical analysis	* 2,3,5	* 1,4,5	* 1	* 2,3	* 1,2

LCR	10	1	10000	10	100
Plasmid	(0.1-100)	(1-100)	(100-1000)	(10- 100)	(1-1000)
	M 10	M 100	M 1000	M 10	M 100
Statistical analysis	* 2	* 1,3,4,5	* 2	* 2	* 2

Synovial fluid was spiked with isolated *C. trachomatis*- elementary bodies (*C. trachomatis *EB) per ml synovial fluid (SF) ranging from 10,000 *C. trachomatis *EB/ml SF to 0.1 *C. trachomatis*-EB/ml SF in 10-fold decreasing concentrations. Five independent repeats of each serial dilution was performed (n = 5) for each DNA extraction method and amplification system evaluated. The range of detection limits of each method is given in brackets, median (M) of serial dilutions given below. Sensitivity was defined as reproducibly detected lowest number of detected *C. trachomatis *EB/ml SF. Statistical analysis: significant results are indicated in order of method compared. Statistical significant results are indicated by *, method compared with is indicated by number (*P *< 0.05). LCR = ligase chain reaction; *omp-1 *= major outer membrane protein.

PCR 1 gave constantly most sensitive detection of *C. trachomatis *EB DNA in combination with all DNA extraction methods applied. None of the other amplification systems allowed higher sensitivity than PCR 1 regardless of the extraction method employed. The DNA extraction methods alkaline lysis and Qiaex II Gel Extraction Kit^® ^+ CTAB allowed almost equal sensitivity limits according to our definition of sensitivity (lowest reproducible detection limit). Because PCR 1 allowed the highest sensitivity with several DNA extraction systems in contrast to the other PCR systems evaluated, in further analysis we restricted comparative detection of different sample preparation procedures based on the results of PCR 1. All internal controls remained negative during PCR analysis.

### Sensitivity of *C. trachomatis*-directed PCR and LCR testing for infected monocytes as a function of template preparation method

*C. trachomatis *EB are the extracellular form of the organism and they possess an extremely durable cell wall. During infection of the joint, the organism is present in the SF and synovial tissue in the intracellular, aberrant body form, which lacks a particular cell wall. To determine whether the methods used for DNA extraction for EB are equally effective in template preparation from intracellular persisting *Chlamydia*, human PBMO persistently infected with *C. trachomatis *were serially diluted in SF. These SF samples spiked with infected PBMO were processed with each of the above listed DNA extraction methods as in the EB studies. The most sensitive detection of chlamydial DNA was performed by DNA extraction by alkaline lysis which repeatedly detected 0.1 *C. trachomatis *PBMO/ml SF. DNA prepared by Qiaex II Gel Extraction Kit^® ^+ CTAB and DNA prepared by the Qiagen tissue kit^® ^allowed detection of 10 *C. trachomatis *PBMO/ml SF in combination with number 1 PCR system. The QIAmp DNA Stool Kit^® ^detected 1 *C. trachomatis *PBMO/ml SF together with PCR system number 1 (Table 3). Chelex^® ^did not achieve sufficient sensitivity in *C. trachomatis *EB serial dilutions and was therefore not performed in the *C. trachomatis *PBMO assays.

**Table 3:** Sensitivity of PCR for the detection of intracellular persisting *Chlamydia trachomatis *in synovial fluid depending on DNA extraction method

Number of serial dilution	1 Alkaline lysis	2 Qiaex II + CTAB gel extraction kit^®^	3 Qiagen Tissue Kit^®^	5 Qiagen Stool Kit^®^
1	1	0.1	10	1

2	0.1	10	100	1

3	0.1	100	1	1

4	1	1	10	1

5	0.1	10	10	1

Sensitivity	0.1 M 0.1	10 M 10	10 M 10	1 M 1

Statistical analysis	* 2, 4	* 1	* 2, 5	* 4

Synovial fluid was spiked with *C. trachomatis *persistently infected peripheral blood monocytes (*C. trachomatis *PBMO) per ml synovial fluid (SF) ranging from 10,000 *C. trachomatis *PBMO/ml SF to 0.1 *C. trachomatis *PBMO/ml SF in 10-fold decreasing numbers. Five independent repeats of each serial dilution were performed (n = 5) for each DNA extraction method. Amplification was performed using system number 1 (*C. trachomatis*-*omp1 *directed PCR). The median (M) of serial dilutions is given below. Sensitivity was defined as reproducibly detected lowest number of detected *C. trachomatis *PBMO/ml SF. Statistical significant results are indicated by *, the method compared with is indicated by number (*P *< 0.05). *omp-1 *= major outer membrane protein.

Alkaline lysis and the Qiagen Stool Kit^® ^allowed significantly lower detection limits of *C. trachomatis *PBMO compared with the Qiagen Tissue Kit^® ^(*P *< 0.05). All controls remained negative during PCR and LCR analysis.

### Influence of storage of DNA on sensitivity of detection limits of *C. trachomatis *EB and *C. trachomatis *PBMO DNA

In routine diagnostic settings, it might become necessary to postpone analysis or reevaluate previously evaluated samples of SF DNA in order to reconfirm or simply repeat results. We therefore addressed the question of how detection limits of chlamydial DNA might change after storage of DNA depending on the different DNA extraction methods applied. To our knowledge, some laboratories performing PCR analysis for routine diagnostic procedures store the extracted DNA at -20°C [25]. Therefore, DNA was stored at -20°C for four months and subjected to the PCR system 1, which was identified as the most sensitive detection system. Detection limits for *C. trachomatis *EB and *C. trachomatis *PBMO decreased dramatically after storage by 10- to 1000-fold. Highest loss of sensitivity was observed after DNA extraction using Qiaex II Gel Extraction Kit^® ^+ CTAB dropping from initial detection limits of 0.1 *C. trachomatis *EB/ml SF and 10 *C. trachomatis *PBMO/ml SF to 1000 *C. trachomatis *EB/ml SF and 1000 *C. trachomatis *PBMO/ml SF after storage of DNA. Detection limits of alkaline lysis dropped from an initial detection of 0.1 *C. trachomatis *EB/ml SF 100 fold to 10 detected *C. trachomatis *EB/ml SF and from 0.1 *C. trachomatis *PBMO/ml SF in immediate analysis to a 100-fold decreased detection rate to 1000 *C. trachomatis *PBMO/ml SF for stored samples (Table 4).

**Table 4:** PCR sensitivities of the different DNA extraction methods detection *Chlamydia trachomatis *EB and *C. trachomatis *PBMO DNA/ml SF using PCR-system 1 for amplification immediately after extraction and post storage at -20°C for four months

Sensitivity achieved with PCR amplification system 1	1 Alkaline lysis immediately	1 Alkaline lysis ps	2 Qiaex II gel extraction kit^® ^+CTAB immediately	2 Qiaex II gel extraction kit^® ^+ CTAB ps	4 Qiagen Tissue Kit^® ^immediately	4 Qiagen Tissue Kit^® ^Ps	5 Qiagen Stool Kit^® ^immediately	5Qiagen Stool Kit^® ^ps
(*C. trachomatis *EB/ml SF)	(0.1 -10) M 1	10 (1-10) M 10	0.1	1000 (1000) M 1000	1 (1-10) M 1	10 (1-10) M 10	1 (1-100) M 100	1 (1-10) M 10

Statistical analysis ps	See Table 3	*	See Table 3	*	See Table 3		See Table 3	

(*C. trachomatis *PBMO/ml SF)	(0.1-1) M 0.1	1000 (1000) M 1000	10 (0.1 - 100) M 10	1000 (1000) M 1000	10 (1-100) M 10	1000 (1000) M 1000	1 (0.1-1) M 1	1000 (1000) M 1000

Statistical analysis ps	See Table 4	*	See Table 4	*	See Table 4	*	See Table 4	*

Statistical analysis post storage (ps) compared with sensitivity results compared with sensitivity achieved after immediate PCR analysis are indicated by *. EB = elementary bodies; M = median; PBMO = peripheral blood monocytes; SF = synovial fluid.

## Discussion

In previous studies we showed that sensitivity of PCR and LCR for *C. trachomatis *in SF and synovial tissue basically depends on the sample preparation as well as the amplification process itself [6,8,9,17]. However, testing for detection of *C. trachomatis *DNA in SF has not yet been standardized accordingly for use in clinical practice. Laboratories employ different in-house methods for preparation of template DNA as well as different amplification systems. This diversity most likely contributes to the variability of positive testing for *C. trachomatis *in clinical SF samples. No national or international reference standards for in-house tests nor commercially available test systems exist to test for *C. trachomatis *DNA in SF. Moreover, the existing in-house laboratory test systems have not yet been evaluated for their feasibility and sensitivity to detect *C. trachomatis *DNA in SF in clinical practice. We therefore analyzed five previously published DNA extraction methods and five amplification systems - four PCR systems and one commercially available LCR - currently used in different laboratories in Europe and the USA for *C. trachomatis *in SF in order to develop a test procedure that would be applicable in the routine diagnostic setting [6,9,17,18,23] The Amplicor Roche^® ^PCR, which when performed in previous studies was less sensitive [8,26] than all other systems, was not included in this study.

We initially compared sensitivities to detect *C. trachomatis *EB DNA serially diluted in SF using the five DNA extractions in combination with the five amplification systems. *C. trachomatis *EB represent the extracellular infectious form of *C. trachomatis *This approach was chosen because *C. trachomatis *EB can be quantified accurately and easily diluted in SF. The Qiaex II Gel Extraction Kit^® ^+ CTAB gave the highest sensitivity to detect *C. trachomatis *EB DNA from SF in combination with the *C. trachomatis omp1 *152 bp PCR. Lower, but still reasonable, sensitivities to detect *C. trachomatis *EB DNA were achieved using alkaline lysis, QIAmp Tissue Kit^® ^and QIAmp Stool Kit^® ^in combination with the same amplification system. The same detection limits were observed using alkaline lysis in combination with the plasmid PCR and the 16s RNA PCR as well as using the Qiagen tissue kit^® ^in combination with the plasmid PCR. All other combinations of DNA extraction methods and amplification systems resulted in lower, non-acceptable sensitivities. *C. trachomatis *EB are known to have a strong cell wall. Therefore, we speculate that the decreased sensitivity to detect *C. trachomatis *EB DNA applying alkaline lysis is due to the fact that the chlamydial cell wall is not easily degraded by this method.

In previous studies we already investigated the sensitivity of the Qiaex II gel extraction kit^® ^in combination with the *C. trachomatis omp1 *152 bp PCR and have demonstrated that the DNA extraction method prior to PCR analysis influences the sensitivity to detect *C. trachomatis *DNA in synovial tissue [9] as well as in SF [17]. In a step further we now evaluated for the first time in a more extensive systematic approach five different DNA extraction methods in combination with five different amplification systems for their sensitivity to detect *C. trachomatis *in SF. In the inflamed joint, *Chlamydia *persists intracellularly in monocytes [4,27], which is the reason why the analysis of *C. trachomatis *EB is not fully comparable with the clinical *in vivo *situation. In order to approach more appropriately the *in vivo *situation we also analyzed for the first time persistently *C. trachomatis-*infected monocytes diluted in SF. PCR and LCR results were thought to give higher sensitivity in these assays because the persisting chlamydial cells in the *C. trachomatis *PBMO are undergoing active, intracellular vegetative growth and lack the strong cell wall characteristics of *C. trachomatis *EB [4,21,27]. Moreover, some monocytes were observed to be infected with more than one *C. trachomatis *(data not shown). But, only DNA extracted by alkaline lysis resulted in higher sensitivity than with isolated EBs. This might be due to the fact that intracellular persisting *Chlamydia *are showing an aberrant gene expression profile [27], which may influence the ease with which DNA extraction methods can release chlamydial DNA. Therefore, the alkaline lysis is superior to other DNA methods to extract DNA from intracellularly persisting *C. trachomatis*.

Altogether, alkaline lysis and Qiaex II gel extraction kit^® ^+ CTAB gave reproducibly the highest detection rates in the *C. trachomatis *EB as well as in the *C. trachomatis *PBMO serial dilution analysis. However, the DNA extracted by either method should be amplified without storage of DNA at a temperature of -20°C because this leads to loss of sensitivity to detect the organism. A storage temperature of -20°C was chosen due to practicability reasons. In our and other laboratories [20,23,27,28], the extracted DNA is stored at this temperature to possibly reamplify the sample DNA in order to confirm results. This observation implies that storage of DNA should be avoided in order to maintain the high sensitivity rates that molecular technology techniques such as PCR and LCR allow. However, future studies have to investigate if storage at different temperatures, i.e. -80°C, or with nitric oxide can preserve the high detection rate.

## Conclusions

In summary, alkaline lysis and the QIAmp gel extraction kit^® ^+ CTAB in combination with the most sensitive *C. trachomatis *- *omp1*- 152 bp - PCR are the most sensitive test systems for detection of chlamydial DNA in *C. trachomatis *SF serial dilutions. However, analysis of SF samples from patients with various rheumatological diseases showed that alkaline lysis has a higher sensitivity to detect *C. trachomatis *DNA from clinical SF samples (data submitted elsewhere). Given its high sensitivity, simplicity, reliability, cost-effectiveness and no requirement of toxic chemicals, the alkaline lysis should to our mind be considered the most feasible detection system of *C. trachomatis *in SF for standardized testing in a clinical practice and to advance the diagnosis of CIA.

## Abbreviations

bp: base pairs; BSA: bovine serum albumin; CIA: chlamydia induced arthritis; EB: elementary bodies; IFU: infection forming units; LCR: ligase chain reaction; MOMP: major outer membrane protein; Omp-1: major outer membrane protein 1; PBMO: peripheral blood monocytes; PBS: phosphate buffered saline; PCR: polymerase chain reaction; ReA: reactive arthritis; SD: standard deviation; SF: synovial fluid.

## Competing interests

The authors declare that they have no competing interests.

## Authors' contributions

JF was responsible for organizational aspects of the study, collection of clinical samples, culture of *C. trachomatis *and performed DNA extraction, PCR analysis and drafted the manuscript. IB and SM performed parts of DNA extraction as well as parts of PCR analysis. Additionally IB performed transportation of samples. HZ and JK conceived of the study and participated in its design and coordination and helped to draft the manuscript. All authors read and approved the manuscript.
